# Biochemical and Structural Characterization of OXA-405, an OXA-48 Variant with Extended-Spectrum β-Lactamase Activity

**DOI:** 10.3390/microorganisms8010024

**Published:** 2019-12-21

**Authors:** Saoussen Oueslati, Pascal Retailleau, Ludovic Marchini, Laurent Dortet, Rémy A. Bonnin, Bogdan I. Iorga, Thierry Naas

**Affiliations:** 1 EA7361 “Structure, dynamic, function and expression of broad spectrum β-lactamases”, Faculty of Medicine of Paris-Sud University, Labex LERMIT, University Paris-Saclay, 94270 Le Kremlin-Bicêtre, France; oueslati.saoussen@gmail.com (S.O.); laurent.dortet@aphp.fr (L.D.); remy.bonnin@u-psud.fr (R.A.B.); 2 Institut de Chimie des Substances Naturelles, CNRS UPR 2301, Labex LERMIT, 91198 Gif-sur-Yvettte, France; pascal.retailleau@cnrs.fr (P.R.); ludovic.Marchini@vinci.fr (L.M.); bogdan.iorga@cnrs.fr (B.I.I.); 3 French National Reference Center for Antibiotic Resistance: Carbapenemase-producing Enterobacteriaceae, 94270 Le Kremlin-Bicêtre, France; 4 Bacteriology-Hygiene unit, Bicêtre Hospital, Assistance Publique/Hôpitaux de Paris, 94270 Le Kremlin-Bicêtre, France

**Keywords:** oxacillinase, OXA-ESBL, carbapenemase, substrate selectivity, beta-lactamase, crystal structure, docking, antibiotic resistance

## Abstract

OXA-48-producing Enterobacterales have now widely disseminated globally. A sign of their extensive spread is the identification of an increasing number of OXA-48 variants. Among them, three are particularly interesting, OXA-163, OXA-247 and OXA-405, since they have lost carbapenem activities and gained expanded-spectrum cephalosporin hydrolytic activity subsequent to a four amino-acid (AA) deletion in the β5–β6 loop. We investigated the mechanisms responsible for substrate specificity of OXA-405. Kinetic parameters confirmed that OXA-405 has a hydrolytic profile compatible with an ESBL (hydrolysis of expanded spectrum cephalosporins and susceptibility to class A inhibitors). Molecular modeling techniques and 3D structure determination show that the overall dimeric structure of OXA-405 is very similar to that of OXA-48, except for the β5–β6 loop, which is shorter for OXA-405, suggesting that the length of the β5–β6 loop is critical for substrate specificity. Covalent docking with selected substrates and molecular dynamics simulations evidenced the structural changes induced by substrate binding, as well as the distribution of water molecules in the active site and their role in substrate hydrolysis. All this data may represent the structural basis for the design of new and efficient class D inhibitors.

## 1. Introduction

The widespread use of antibiotics led to the emergence of carbapenem resistance in Gram-negative bacteria and became a real clinical concern. This resistance is mostly due to the production of carbapenem-hydrolyzing β-lactamases, carbapenemases that belong to Amber class A (KPC), B (NDM, VIM, and IMP), or D (OXA-48 and its variants) [1]. One of the main carbapenemases is the class D β-lactamase OXA-48, which represents a major public health threat because of its rapid spread worldwide [2,3,4]. Although OXA-48 hydrolyzes penicillins at high level, it hydrolyzes carbapenems at a low level and shows very weak activity against expanded-spectrum cephalosporins. However, when associated to impaired outer-membrane permeability and an ESBL, OXA-48-producers may turn into deadly bacteria, as only limited antibiotic choices are left for treating serious infections with these bacteria [5].

**Figure 1:**
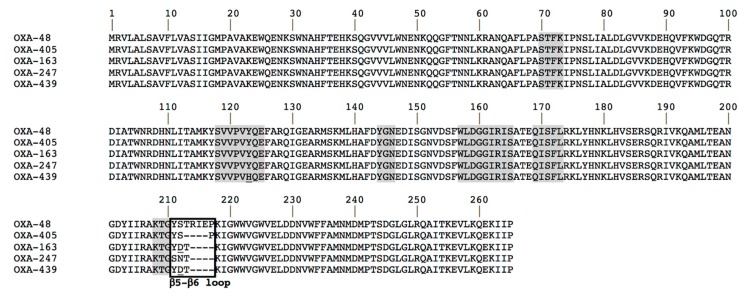
Amino acid sequence alignment of OXA-48 variants. Amino acid motif that are well conserved among class D lactamases are indicated by gray shading, and the black frame corresponds to the β5–β6 loop. Numbering is according to OXA-48 sequence.

The OXA-48 β-lactamase was initially identified from a *Klebsiella pneumoniae* isolate in Istanbul (2004) [4]; it then rapidly spread throughout the Mediterranean area, the Middle East, and Europe [6] and became an increasing threat. Since OXA-48’s first identification, several OXA-48 variants have been reported worldwide [7,8]. All these enzymes, except OXA-163, OXA-247, and OXA-405, have similar hydrolytic profiles as OXA-48: a high level of hydrolysis of penicillin, a low level of carbapenem hydrolysis, but no significant hydrolysis of broad-spectrum cephalosporins, such as ceftazidime [9]. OXA-163 (identified from *K. pneumoniae* and *Enterobacter cloacae* isolates in Argentina and in Egypt) [10,11], OXA-247, a point mutant derivative of OXA-163 from Argentina [12], OXA-439, another point mutant derivative of OXA-163 (unpublished, KP727573.1), and OXA-405 (identified from *S. marcescens* in France) [13] differ by a four amino-acid deletion (213-TRIE-217 for OXA-405; 214-RIEP-217, plus a substitution S212D for OXA-163, Y124H, and S212D for OXA-439, and YS211-212SN for OXA-247), located in the β5–β6 loop, which results in a modified β-lactam spectrum of hydrolysis (Figure 1). Indeed, analysis of the hydrolytic properties of OXA-163 and OXA-405 showed an ESBL-type profile, that they hydrolyze cefotaxime and cephalothin efficiently, are partially inhibited by clavulanate, and lack significant carbapenem hydrolysis [13]. The aim of this study was to investigate the mechanisms responsible for this peculiar substrate specificity of OXA-405, using biochemical and structural tools, and to compare the resulting structure and the hydrolytic profile with those of OXA-163 and OXA-48.

## 2. Materials and Methods

### 2.1. Bacterial Strains

The clinical strain *S. marcescens* expressing the OXA-405 β-lactamase was used for the cloning of the *bla*_OXA-405_ gene [13]. *E. coli* TOP10 (Invitrogen, Saint-Aubin, France) was used for cloning, and *E. coli* BL21 (DE3) (Novagen, Fontenay-sous-Bois, France) was used for overexpression experiments.

### 2.2. PCR, Cloning, Expression, and DNA Sequencing

Whole-cell DNA of *S. marcescens* [13] isolates producing OXA-405 was extracted, using the QIAamp DNA mini kit (Qiagen, Courtaboeuf, France) and used as a template to amplify the *bla*_OXA-405_ gene. The sequences without the peptide signal (predicted by SignalIP 4.1 Server) of *bla*_OXA-405_ gene, encoding for the mature protein from amino acid K23 to P261, were obtained by PCR amplification, using primers OXA_23-256_NdeI (5′-aaaaaCATATGaaggaatggcaagaaaacaaa-3′), which included an *Nde*I restriction site and the reverse primer OXAXhoI-Δstop (5′-aaaaaCTCGAGggggaataatttttcctgtttgag-’3), which included an *Xho*I restriction site and a deletion of the stop codon of the gene to allow the expression of an C_Term His tag. Then, PCR product was cloned into pET41b vector (Invitrogen^®^, Life Technologies, Cergy-Pontoise, France), using *Nde*I and *Xho*I restriction enzymes, to obtain a C-Term His_8_-tag. The accuracy of the recombinant plasmid was verified by sequencing, using a T7 promoter and T7 terminator with an ABI Prism 3100 automated sequencer (Applied Biosystems, Thermo Fisher Scientific, Les Ulis, France). The nucleotide sequences were analyzed by using software available at the National Center for Biotechnology Information website (http://www.ncbi.nlm.nih.gov).

### 2.3. Protein Purification

The recombinant plasmid pET41b-OXA-405_23-265 HIS_ was transformed into *E. coli* BL21 (DE3), and an overnight culture was used to inoculate 2 L of Luria Bertani medium broth containing 50 μg/mL of kanamycin. Bacteria were cultured at 37 °C, until an OD of 0.6 at 600 nm was reached. The expression of the β-lactamase genes was carried out at 37 °C for 3 h, with 1 mM of IPTG as an inducer. Cells were pelleted by centrifugation, at 6000× g for 15 min, then resuspended with 25 mM of phosphate sodium pH 7.4, 300 mM of K_2_SO_4_, and 10 mM of imidazole. Bacterial cells were disrupted by sonication, and the bacteria debris was removed by 2 centrifugations: first at 10,000× *g* for 1 h at 4 °C, and then the supernatants obtained were centrifuged at 96,000× *g* for 1 h at 4 °C. The soluble fraction was filtered and then passed through a HisTrap^TM^HP column (GE Healthcare^®^, Velizy-Villacoublay, France). The OXA-405_His8_ protein was eluted, using elution buffer (25 mM of phosphate sodium pH 7.4, 300 mM of K_2_SO_4_, and 500 mM of imidazole). The eluted protein was concentrated by using Vivaspin 20 (10 000 MWCOPES Sartorius^®^, Aubagne, France) and then dialyzed against 0.1 M of HEPES (pH 7.5) buffer. The protein purity, estimated by SDS–PAGE, was more than 99%, and the pooled fractions were dialyzed against 10 mM of Tris-HCl pH 7.6 and concentrated, using Vivaspin columns. Protein concentration was determined by Bradford Protein assay (Bio-Rad, Marnes-La-Coquette, France).

### 2.4. Steady State Kinetic Determinations

Purified β-lactamase was used for kinetic measurements, which were determined in 100 mM of Tris-H_2_SO_4_ and 300 mM of K_2_SO_4_ (pH 7), and antibiotics were purchased from Sigma-Aldrich (Saint-Quentin Fallavier, France) [9]. The *k*_cat_ and *K*_m_ values were determined by analyzing β-lactam hydrolysis under initial-rate conditions, with an ULTROSPEC^®^ 2000 model UV spectrophotometer (GE Healthcare, Velizy-Villacoublay, France) and SWIFT II software (GE Healthcare, Velizy-Villacoublay, France), using the Eadie–Hofstee linearization of the Michaelis–Menten equation. The concentration that reduced the level of hydrolysis by 50% (IC_50_) was determined in a buffer comprising 100 mM of Tris-H_2_SO_4_ and 300 mM of K_2_SO_4_ (pH 7), with 100 μM of benzylpenicillin as a reporter substrate. The enzymes were incubated with different concentrations of inhibitors, for 3 min, before the kinetic parameter determination [9].

### 2.5. Protein Crystallization and X-Ray Crystallography

Crystallization conditions using commercial kits (Classics, PEGs I and II, and AmSO4 suites from Qiagen, Courtaboeuf, France) were screened in sitting-drop vapor diffusion experiments, using a Cartesian nanodrop robot (Genomic Solutions, Ann Harbor, MI, USA) on the I2BC Crystallization Platform (CNRS, Gif-sur-Yvette, France). OXA-405 at 33 mg/mL concentration was crystallized over a reservoir containing 2.2 M of ammonium sulfate and 0.2 M of sodium fluoride. Crystals were transferred to a cryoprotectant solution (mother liquor supplemented with 20% glycerol) and flash-frozen in liquid nitrogen. Diffraction data were collected at 100 K, to a resolution of 2.26 Å, using a RIGAKU MicroMax™ 007 HF rotating copper anode with OSMIC VariMaxHF mirrors and an MAR345 image plate detector, at the Institut de Chimie des Substances Naturelles (CNRS, Gif-sur-Yvette, France). Diffraction intensities were integrated with the program XDS [14].

The dimer structure of OXA-405 was solved by molecular replacement with Molrep [15], using the monomer moiety of the OXA-48 structure (Protein Data Bank code 3HBR [16]) as a search model. Model refinement was performed with BUSTER-TNT [17]. Electron density maps were evaluated, using COOT [18] for manual refinement. The maps revealed clear density for the carboxylated lysine-73 residues. In contrast, the side chain of the N-ter Met-22 residues, as well as the C-ter 6xHis-tag, was unseen. Refinement details of the structure are shown in Table 3. Molecular graphics images were generated using UCSF Chimera version 1.10.2 (https://www.cgl.ucsf.edu/chimera/) [19].

### 2.6. Structure Analysis, Docking, Molecular Dynamics, and Water Network Analysis

OXA-405 structural analysis and comparison with other crystal structures were performed with UCSF Chimera package [19]. Covalent docking calculations were performed, using the GOLD software, version 5.2 (CCDC suite) [20]. Ligand structures were generated with 3D Structure Generator CORINA Classic (Molecular Networks GmbH, Nuremberg, Germany). Molecular dynamics simulations of OXA-405 were performed with Gromacs version 4.6 [21], using the OPLS-AA force field [22]. HOP software version 0.4.0 alpha 2 (https://github.com/Becksteinlab/hop) [23] was used for water molecule dynamics analysis.

### 2.7. PDB Deposition

The crystallographic structure of OXA-405 was deposited into the Protein Data Bank (PDB) [24], accession code 5FDH.

## 3. Results

### 3.1. Biochemical Properties of OXA-405

To confirm previous findings based only on specific activities [13], and to understand the structural features explaining the differences observed with OXA-48, we purified OXA-405, determined the 3D structure, and determined the steady-state kinetic parameters for several clinically relevant substrates (penicillin, cephalosporins, and carbapenems) and compared them to those of OXA-163 and OXA-48 (Table 1). For OXA-48, the highest catalytic efficiency was observed for amoxicillin hydrolysis (2400 mM^−1^ s^−1^) (Table 2). As observed by others, carbapenems are among the preferred OXA-48 substrates, with the highest catalytic efficiency (*k*_cat_/*K*_m_) observed for the hydrolysis of imipenem (370 mM^−1^ s^−1^) [9,16]. Compared to imipenem, the OXA-48 *k*_cat_/*K*_m_ value is 59-fold smaller for meropenem and 284-fold smaller for ertapenem hydrolysis because of a reduction in the turnover number (*k*_cat_). In addition, the early cephalosporin, cephalothin, is hydrolyzed with a *k*_cat_/*K*_m_ slightly higher than that of the oxyimino cephalosporin cefotaxime mainly because of a lower *K*_m_ value. Finally, the steady-state kinetic analysis indicates OXA-48 does not hydrolyze the bulky oxyimino–cephalosporin ceftazidime, as previously suggested. The substrate profile of OXA-405 is substantially different from that of OXA-48, but quite similar to that of OXA-163. Unlike OXA-48, OXA-405 hydrolysis preferentially cephalosporins (Table 1). The highest *k*_cat_/*K*_m_ values were observed for benzylpenicillin (667 mM^−1^ s^−1^), ampicillin (137 mM^−1^ s^−1^), cephalothin (444 mM^−1^ s^−1^), and cefotaxime (26 mM^−1^ s^−1^). Ceftazidime was hydrolyzed with the lowest *k*_cat_/*K*_m_ (0.7 mM^−1^ s^−1^) among the cephalosporins tested because of a high *K*_m_ value. Nevertheless, OXA-405 does hydrolyze ceftazidime. The carbapenemase activity of OXA-405 is attenuated largely because of a reduction in the turnover number and of the affinity for carbapenems, except for ertapenem; the carbapenemase activity was nevertheless higher for OXA-405 as compared to OXA-163.

This leads to a decrease of 1850-fold in the catalytic efficiency of OXA-405 for imipenem in comparison to that of OXA-48, while, for OXA-163, a 6166-fold decrease was observed. However, it is noteworthy that the catalytic efficiencies of OXA-405 are higher for ertapenem and imipenem as compared to meropenem. Although the reason for this cannot be known without further structural data, it is possible that the smaller size of imipenem in comparison to meropenem and ertapenem allows it to fit better into the active site of OXA-48, leading to a higher rate of turnover of imipenem by OXA-48, which is then lost due to the active-site expansion that occurs in OXA-405, as described below.

In summary, the results confirm the ability of the OXA-48 active site to accommodate carbapenem substrates, particularly imipenem, while it is unable to hydrolyze ceftazidime, a bulkier oxyimino-cephalosporin. Our results confirm, that the four AA deletion in the β5–β6 loop observed in OXA-405 is responsible for a drastic loss of carbapenemase activity, particularly for imipenem, and a gain of cephalosporinase activity with the ability to hydrolyze cefotaxime and ceftazidime. The hydrolytic properties of OXA-405 make it more similar to an Amber class A extended-spectrum β-lactamase. Therefore, the 213-TRIE-217 alters the substrate specificity of OXA-405. This finding is consistent with previously published kinetic data showing a significant increase in the level of ceftazidime hydrolysis compared to that of OXA-48. Additionally, two other members of the OXA-48-like β-lactamases, OXA-247 and OXA-163, which differ from OXA-48 by a four amino acid deletion, 214-RIEP-218 and 214-RIEP-217 deletion and S212D substitution, respectively, have significantly lowered activity toward carbapenem substrates as compared to that of OXA-48 [16]. Determination of IC_50_ (Table 2) showed that OXA-405, as OXA-163 and OXA-48, can be inhibited by clavulanic acid (6 μM), tazobactam (1.8 μM), and NaCl (40 × 10^3^ μM).

In a general way, OXA-405 and OXA-163 have similar catalytic efficiency values for penicillin, which are lower than those of OXA-48. Concerning cephalosporins, the three enzymes have nearly the same catalytic efficiency for cephalothin, however, there is a difference for the hydrolysis of cefotaxime: value of OXA-405 (26 mM^−1^ s^−1^) is two-fold higher than OXA-48 (10 mM^−1^ s^−1^) but nine-fold lower than that of OXA-163 (230 mM^−1^ s^−1^). The *k*_cat_/*K*_m_ values for ceftazidime of OXA-405 (0.7 mM^−1^ s^−1^) and OXA-163 (1.3 mM^−1^ s^−1^) are low, but significant to confer ceftazidime resistance in bacteria expressing these enzymes, which is not the case for OXA-48. The fact that OXA-163 hydrolyses better cefotaxime and ceftazidime as compared to OXA-405 is likely related to the two-point mutations between these two enzymes.

### 3.2. X-Ray Crystallography

The crystal structure of OXA-405 refined at 2.26 Å presents the typical class D fold with an α-helical region and a mixed α-helix/β-sheet region, with 96% of all residues inside the favored regions of the Ramachandran plot, 4% in the allowed regions, and no outliers (Table 3).

**Figure 2:**
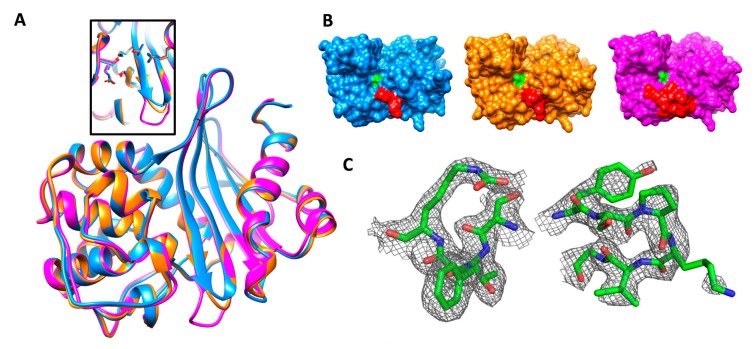
(**A**) Superposition of crystal structures of OXA-405 (blue, PDB 5FDH), OXA-163 (orange, PDB 4S2L) and OXA-48 (magenta, PDB 3HBR), with an insert showing the almost perfect superposition of key binding site residues of the three enzymes. (**B**) Surface representation of the three enzymes with the same color scheme as above. Ser70 and the β5–β6 loop are colored in green and red, respectively. (**C**) Residues from the STFK motif (left) and the β5–β6 loop (right) in stick representation, with a 2Fo-Fc map contoured at 0.8 σ.

The asymmetric unit contains two protein chains, A and B, modeled with 246 residues each. The OXA-405 structure has a Cα RMSD of 0.52 Å on 236 residues compared to the OXA-48 structure (PDB code 3HBR [16]) and 0.37 Å on 237 residues compared to OXA-163 (PDB code 4S2L [25]). The conformations of residues from active sites are very similar (Figure 2A), and the only difference between these three structures is the shorter β5–β6 loop in OXA-405 and OXA-163, due to the four-residue deletion in this loop (Figure 2A), which induces important changes in the corresponding active sites (Figure 2B). Clear electron density is observed for both backbones and side chains, including the active site and the β5–β6 loop, with the exception of side chains of Lys218 (OXA-48 numbering) from the β5–β6 loop (Figure 2C) and of Met22 (not shown).

### 3.3. Structural Analysis of Covalent Protein-Ligand Complexes

**Figure 3:**
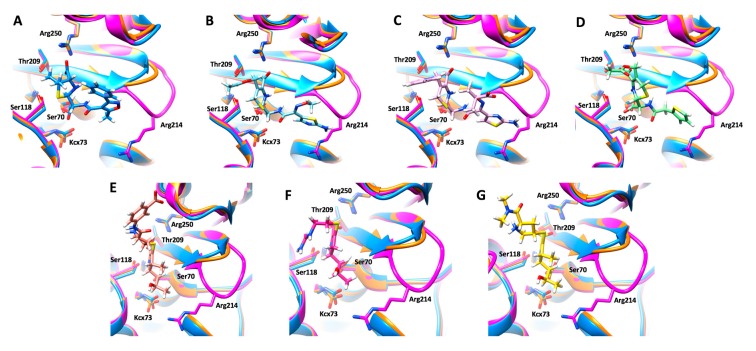
Covalent docking poses of oxacillin ((**A**), blue sticks), cefotaxime ((**B**), cyan sticks), ceftazidime ((**C**), pink sticks), cephalotin ((**D**), green sticks), ertapenem ((**E**), orange sticks), imipenem ((**F**), red sticks) and meropenem ((**G**), yellow sticks), in the active site of OXA-405 (blue). Superposed OXA-163 (orange) and OXA-48 (magenta) are also shown for comparison. Key residues of the active sites are represented as sticks and labelled using the OXA-48 numbering.

A molecular modelling study was performed to identify the structural determinants that could explain the differences between the hydrolytic profiles of OXA-405 and OXA-48. Covalent docking calculations were performed with β-lactam antibiotics belonging to the penicillin (oxacillin), cephalosporin (cefotaxime, ceftazidime, and cephalothin), and carbapenem (ertapenem, imipenem, and meropenem) families into the active site of OXA-405 (Figure 3).

The binding mode of oxacillin is not influenced by the four-residues’ deletion in the β5–β6 loop due to the relatively small size of the R2 substituent in the penicillin family (Figure 3A), which is in agreement with similar *K*_m_ values determined for OXA-405 and OXA-48 (Table 1). In contrast, the R2 substituent of cephalosporins has unfavorable steric clashes with Arg214 in OXA-48 that are not observed in OXA-405, where this residue is absent and the β5–β6 loop is shorter (Figure 3B–D). These structural differences explain the systematically lower *K*_m_ values of cephalosporins in the complex with OXA-405 compared with those of OXA-48 (Table 1). On the other hand, the R2 substituent in carbapenems is quite small compared to other β-lactams, and, in this case, the protein-ligand complexes obtained by covalent docking (Figure 3E–G) cannot fully explain the higher *K*_m_ values determined with imipenem and meropenem, but not with ertapenem, in the complex with OXA-405 as compared with OXA-48 (Table 1). These differences would likely come from contrasting levels of flexibility of the β5–β6 loop in OXA-405 and OXA-48, due to the absence in the former case of the stabilizing interaction between Arg214 (which is part of the four-residues deleted in the β5–β6 loop) and Asp159 from the Ω loop.

### 3.4. Molecular Dynamics and Water Network Analysis

**Figure 4:**
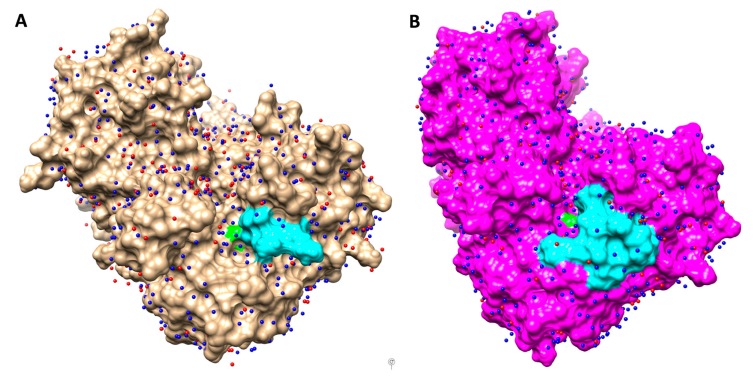
Crystallographic water molecules (red) and stabilized water molecules computed with HOP (blue) represented on the surfaces of OXA-405 ((**A**), brown) and OXA-48 ((**B**), magenta). Ser70 and β5-β6 loop are colored in green and cyan, respectively.

Molecular dynamics (MD) simulations of OXA-405 and OXA-48 dimers (50 ns each) that were carried out by using Gromacs [21], with the OPLS-AA force field [22], confirmed the stability of these systems (RMSD values of 2 Å or less for the entire length of the simulation) and constituted the input data for the water network analysis, using the HOP software [23]. The stabilized water molecules identified in this way on the protein surfaces of OXA-405 and OXA-48 are shown in Figure 4.

These stabilized waters are more numerous than the crystallographic waters determined for these two proteins, highlighting the superiority of the in-silico approach, which is not dependent on the crystal structure resolution and takes into account the protein flexibility. In some cases, the positions of waters determined using these two approaches were quite similar, emphasizing the convergence of these two methods and the possible use of MD simulations to improve the refinement of crystallographic structures [26].

## 4. Discussion

OXA-48-producing Enterobacteriaceae are now endemic in the Middle East, North Africa, and India [7]. Since its first description, many variants have been identified [8]. Three of them are interesting—OXA-405, OXA-163 [10], and OXA-247 [12]—because they have a significant activity toward expanded-spectrum cephalosporins but a very weak activity toward carbapenems. In this study, we characterized the biochemical and structural properties of OXA-405, which has a four amino-acid deletion 213-TRIE-217 compared to OXA-48. The steady-state kinetics of OXA-405 revealed that the deletion conferred a decrease of the catalytic efficiency for carbapenems, particularly imipenem (with a decrease of 1850-fold), but gains the ability to hydrolyze ceftazidime as compared to OXA-48. This data show that the 213-TRIE-217 deletion alter the substrate specificity of the enzyme. This finding is in agreement with previously published kinetics data of OXA-163, which has approximatively the same deletion 214-RIEP-217, plus a substitution S212D [9]. Additionally, another member of the OXA-48-like β-lactamases, OXA-247 [12,27], which differs from OXA-48 by another four amino-acid deletion 214-RIEP-218, has significantly lowered activity toward carbapenem substrates as compared to OXA-48. The IC50 values show that OXA-405, similarly to OXA-163, is susceptible to the inhibition by class A inhibitors (tazobactam and clavulanic acid).

The crystal structure of OXA-405 showed that the 213-TRIE-217 deletion did not modify the overall structure of the protein (Figure 2A) but resulted in an important decrease of the size of the β5–β6 loop, with important consequences on the overall shape of the binding site (Figure 2B). The Lys218 is flexible, as shown by the reduced electron density observed on this side chain (Figure 2C). The covalent docking calculations showed that Arg214 plays a key role in defining the hydrolysis profile. In OXA-48, this residue blocks the binding of cephalosporins through unfavored steric clashes with their bulky R2 substituents, whereas the carbapenems are accommodated into a specific conformation of the active site stabilized by the ionic interaction between Arg214 and Asp159 [27]. OXA-405 features a more open conformation of the active site due to the absence of Arg214 and its stabilizing interaction with Asp159, as well as the smaller size of the β5–β6 loop. This leads to an increased affinity for cephalosporins and a decreased stabilization of carbapenems in the active site, thus explaining the important differences in affinity for these substrates observed between OXA-48 and OXA-405. Overall, all these observations may constitute the basis for the development of efficient new inhibitors targeting all OXA-48-like β-lactamases, and even the false carbapenemases like OXA-163, OXA-247, and OXA-405.

## 5. Conclusions

OXA-405 is a peculiar OXA-48-like enzyme, as it has lost carbapenem activities and gained expanded-spectrum cephalosporin hydrolytic activity subsequent to a 4 amino-acid (AA) deletion in the β5–β6 loop. Kinetic parameters confirmed that OXA-405 behaves more like an ESBL ß-lactamase, rather than a carbapenemase. Molecular modeling techniques and 3D structure determination show that the overall dimeric structure of OXA-405 is very similar to that of OXA-48, except for the β5–β6 loop, which is shorter for OXA-405, suggesting that the length of the β5–β6 loop is critical for substrate specificity. Covalent docking with selected substrates and molecular dynamics simulations evidenced the structural changes induced by substrate binding, as well as the distribution of water molecules in the active site and their role in substrate hydrolysis. All this data may represent the structural basis for the design of new and efficient class D inhibitors. Our findings provide a molecular basis for the hydrolysis of ceftazidime by OXA-405 and, more broadly, illustrate further how minor sequence changes can profoundly alter the structure of the active site and thereby affect the substrate profile of an enzyme. Furthermore, our results should allow us to better understand the potential of class D carbapenemases, to extend their spectrum, and thus evaluate the potential threat to public health.

## Figures and Tables

**Table 1:** Steady-state kinetic parameters ^a^ for hydrolysis of β-lactam substrates by OXA-405, OXA-163, and OXA-48 β-lactamases.

	*K*_m_ (μM) ^a^			*k*_cat_ (s^−1^)			*k*_cat_*/K*_m_ (mM^−1^ s^−1^)		
Substrate	OXA-405	OXA-163	OXA-48	OXA-405	OXA-163	OXA-48	OXA-405	OXA-163	OXA-48
Benzylpenicillin	18	13	ND ^b^	12	23	ND	667	1800	ND
Ampicillin	212	315	395	29	23	955	137	70	2400
Oxacillin	69	90	95	19	34	130	275	370	1400
Temocillin	NH ^c^	NH	45	NH	NH	0.3	ND	ND	6.6
Cephalothin	18	10	195	8	3	44	444	300	225
Cefotaxime	369	45	>900	9.7	10	>9	26	230	10
Ceftazidime	>1000	>1000	NH	0.7	8	NH	0.7	3	NH
Imipenem	532	520	13	0.1	0.03	4.8	0.2	0.06	370
Meropenem	>2000	>2000	11	0.1	>0.1	0.07	0.09	0.03	6.2
Ertapenem	88	130	100	0.04	0.05	0.13	0.4	0.3	1.3

^a^: data are the mean of three independent experiments; standard deviations were within 10% of the mean; ^b^: ND, not determined; ^c^: NH, no detectable hydrolysis was observed with 1 mM of purified enzyme and 500 μM of substrate. Data for OXA-163 are from Oueslati et al. [9] and OXA-48 from Docquier et al. [16].

**Table 2:** Fifty percent inhibitory concentration (IC50) of clavulanic acid and tazobactam for β-lactamases OXA-405, OXA-163, and OXA-48 ^a^.

Inhibitor	IC50 (μM)		
	OXA-405	OXA-163	OXA-48
Clavulanic acid	6	13.4	28.5
Tazobactam	1.8	0.75	20
NaCl	40 × 10^3^	97 × 10^3^	35 × 10^3^

Note: ^a^ data are the mean of three independent experiments; standard deviations were within 10% of the mean. Data for OXA-163 from Stojanoski et al. [25].

**Table 3:** X-ray data collection and refinement statistics.

	OXA-405
Protein Data Bank code	5FDH
wavelength (Å)	1.54187
Space group	P4_3_2_1_2
Asymmetric unit	1 dimer
Unit cell (Å)	
*A*	90.40
*B*	90.40
*C*	172.63
α (deg)	90.0
β (deg)	90.0
γ (deg)	90.0
Resolution (Å)	13.12–2.26
Observed reflections	35,642 (4116) ^a^
Unique reflections	10,205 (1346)
Completeness (%)	98.0 (90.3)
*I*/σ (*I*)	18.9 (4.6)
*R*_sym_ (%)	9.7 (46.8)
*R*_cryst_ (%)	17.5
*R*_free_ (%)	21.3
no. of nonhydrogen atoms	4482
Protein	3952
heterogen	530
Waters	434
no. of protein residues	484
no. of ligands	8 SO4, 3 GOL
Root mean square deviation	
Bond lengths (Å)	0.010
Bond angles (deg)	1.10
Ramachandran	
favored (%)	96
outliers (%)	0
Mean *B* value (Å^2^)	
Protein	38.2 (chain A), 39.1 (chain B)
Solvent	46.1 (SO4) 55.2 (GOL), 45.9 (HOH)

^a^ Numbers in parentheses represent values in the highest resolution shell: 2.26–2.40 Å (OXA-405).
